# Acupuncture treatment for post-stroke depression: Intestinal microbiota and its role

**DOI:** 10.3389/fnins.2023.1146946

**Published:** 2023-03-21

**Authors:** Hailun Jiang, Shizhe Deng, Jieying Zhang, Junjie Chen, Boxuan Li, Weiming Zhu, Menglong Zhang, Chao Zhang, Zhihong Meng

**Affiliations:** ^1^National Clinical Research Center for Chinese Medicine Acupuncture and Moxibustion, Tianjin, China; ^2^Graduate School, Tianjin University of Traditional Chinese Medicine, Tianjin, China; ^3^First Teaching Hospital of Tianjin University of Traditional Chinese Medicine, Tianjin, China

**Keywords:** depression, intestinal microbiota, stroke, inflammation, immune response

## Abstract

Stroke-induced depression is a common complication and an important risk factor for disability. Besides psychiatric symptoms, depressed patients may also exhibit a variety of gastrointestinal symptoms, and even take gastrointestinal symptoms as the primary reason for medical treatment. It is well documented that stress may disrupt the balance of the gut microbiome in patients suffering from post-stroke depression (PSD), and that disruption of the gut microbiome is closely related to the severity of the condition in depressed patients. Therefore, maintaining the balance of intestinal microbiota can be the focus of research on the mechanism of acupuncture in the treatment of PSD. Furthermore, stroke can be effectively treated with acupuncture at all stages and it may act as a special microecological regulator by regulating intestinal microbiota as well. In this article, we reviewed the studies on changing intestinal microbiota after acupuncture treatment and examined the existing problems and development prospects of acupuncture, microbiome, and poststroke depression, in order to provide new ideas for future acupuncture research.

## 1. Introduction

The most common neuropsychological disorder after stroke is depression, which can occur at any point during the process. It is estimated that more than one-third of stroke survivors experience post-stroke depression (PSD) (Frank et al., 2022), which is a global public health issue that requires urgent attention in national health policy. Depression ranks as one of the leading causes of disability worldwide and contributes significantly to the global burden of disease, according to the World Health Organization (WHO). Additionally, treatment options recommended by the WHO report include, but are not limited to, psychotherapy and/or antidepressants, the most classic of which are tricyclic antide pressive agents (TcAs) and selective serotonin reuptake inhibitors (SSRIs) (Li et al., 2022a). Drugs, however, have inherent side effects, such as the development of drug resistance, and often reported adverse effects including sexual dysfunction and gastrointestinal symptoms, neuropsychiatric symptoms, and other systemic symptoms (Anagha et al., 2021). Throughout the years, medical treatment patterns have gradually changed, patients’ vital interests are better served by improving the safety of therapeutic measures while pursuing curative effects. When it comes to treating PSD comprehensively, acupuncture has important advantages as an ideal “green treatment.” In recent years, more and more standardized clinical trials have shown acupuncture not only promotes nerve function recovery after stroke, but also significantly benefits in treating patients’ depression symptoms as well as improving their quality of life after stroke, when comparing with drugs, acupuncture offers better biosafety and socioeconomic benefits in the treatment of PSD (Hang et al., 2021; Wang Z. et al., 2021).

In addition to the combination of neurological and psychiatric symptoms, it is also common for PSD patients to have abnormal digestive tract function (Jiang, 2022). Researchers have found a significant difference between patients with PSD and those without PSD in terms of gut microbial community and metabolites (Jiang et al., 2021; Zhong et al., 2022). Therefore, it is possible that reasonable gut microbiome composition may play a significant role in maintaining healthy metabolism, and it is increasingly being recognized that depression can be treated with direct changes in intestinal microbiota composition (such as prebiotic intake and fecal microbiota transplantation) (Evrensel and Tarhan, 2021). Studies have shown that mood and behavior are controlled and affected by intestinal flora through neuroimmune mechanisms and nutritional metabolism, whereas unbalanced gut flora can cause mental illness (Liang et al., 2018b; Waclawiková and El Aidy, 2018). Psychoneurotic symptoms in rats can be significantly improved by acupuncture, which may be related to rebalancing the intestinal flora (Li et al., 2021; Xian et al., 2022).

Consequently, maintaining the balance of intestinal microbiota is expected to be a potential target for PSD with acupuncture. Moreover, bibliometric analysis shows that the number of studies focusing on intestinal flora has been increasing over the past 10 years, indicating that acupuncture regulation of intestinal microbiota is a promising research area (Zhang et al., 2022a). In this article, an overview of the relationship between acupuncture and intestinal flora, the relationship between intestinal flora and PSD, the effect and mechanism of acupuncture on the intestinal flora in preventing and treating PSD were provided in an attempt to provide new ideas and targets for studies of traditional Chinese medicine in the treatment of PSD, and we hope to provide some assistance in the decision-making process for acupuncture PSD treatment in the future.

## 2. The relationship between acupuncture and intestinal microbiota

Human-related microbial communities are mainly found in the large intestine, which mainly composed of prokaryotes (like bacteria), eukaryotes (like fungi and parasites), and viruses (Li et al., 2022b). It is estimated that the total number of bacteria colonized in the human intestinal tract is about 10^13^–10^14^, most of which are *Bacteroides* and Firmicutes and the amount of bacteria is about 10 times as much as the total number of cells in the human body and their number of genes is 100 times higher than the human genome (Gill et al., 2006). Although it only weighs 1–1.5 kilograms total, it plays an important role in maintaining the dynamic balance of the internal environment and promoting human health (Bäckhed et al., 2005; Borrel et al., 2020).

Microecological community’s succession are intricately intertwined with intestinal various physiological and pathological processes in the body, especially metabolism and immunity (Lynch and Hsiao, 2019). As soon as intestinal microecology homeostasis is disrupted (e.g., reduced richness, dysfunctional microflora, an interference with metabolism or microflora translocation, etc.), it stimulates immune response disorders through different mechanisms and destroys the host immune system as a result and various immune-mediated inflammatory responses will occur, endangering human health (Ruff et al., 2020).

In different disease states, acupuncture can effectively regulate intestinal microbiota, making it a useful microecological regulator. According to existing studies, acupuncture can treat diseases by conducting information exchange between immune-neuro-endocrine-microbial metabolism through brain-intestinal interaction (Xu and Lu, 2020). For example, acupuncture affects the abundance and structure of intestinal bacteria, balancing the number and proportion of probiotics and pathogens in the host body (Xie et al., 2020; Wang J. M. et al., 2021; Wang T. Q. et al., 2021; Li et al., 2022d). In turn, acupuncture reverses a variety of intestinal flora metabolic disorders caused by various diseases by restoring the function and metabolic pathway of key metabolites in human body (Xu et al., 2017; Si et al., 2022). In the restoration of human health, acupuncture plays a crucial role. However, research on acupuncture’s regulation of intestinal flora is still limited in the domestic and overseas, facing the problem of limited scope and insufficient depth.

## 3. The relationship between intestinal flora and PSD

As a secondary to stroke, PSD is characterized by mental and emotional disorders, as well as insomnia, low mood, loss of interest, and loss of appetite and serious people will even exhibit concerning behavioral and psychological characteristics, such as fantasy, delusion, world-weariness, suicide (Wijeratne et al., 2022). Currently, there is no clear pathogenesis for PSD. Current mainstream views is that depression consists of a number of interactions between neurobiology and social psychology and other factors (Li et al., 2022c), and cerebral vascular disease may be a predisposing factor or a motivating factor for depression (Jeon and Kim, 2018).

Depression and digestive problems have strong comorbidities, and many patients with depression go to the hospital for the first attendance usually due to difficult-to-treat gastrointestinal conditions (Liang et al., 2018a; Jiang, 2022). With the deepening of research, researches have gradually illuminated the close connection between psychological factors and gastrointestinal disease over the years. In a meta-analysis, depression was found in 22–38% of patients with irritable bowel syndrome (Hu et al., 2021), and in a cohort study, depression was a comorbid condition for 40.1% of those with inflammatory bowel disease (Lewis et al., 2019). Not only a high prevalence of depression in digestive diseases, but vice versa as well. In another study, researchers found that the rate of gastrointestinal abnormalities among patients with depression was significantly higher than that of those without depression and compared with patients with depression alone, those with depression combined with gastrointestinal symptoms had a more severe depression (Fang and Li, 2022). As a result of these findings, researchers are now paying more attention to gut-brain connection.

**FIGURE 1:**
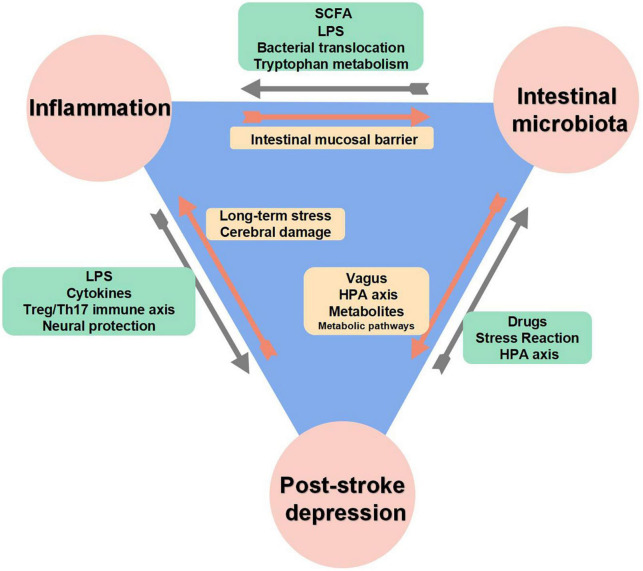
The interaction between inflammation, intestinal microbiota and post-stroke depression (PSD).

According to some available research, changes in the composition of the gut microbiome were strongly associated with the severity of PSD, as shown in Figure 1 (Jiang et al., 2015; Ye et al., 2021). A neuro-endocrine-immune network exists between the brain and gut flora, called the brain-gut axis, which communicates two-way signals, and the gastrointestinal tract is closely linked to the brain mainly through neural and humoral pathways, allowing this network to circulate and reinforce each other (Begum et al., 2022; Han et al., 2022). Studies have shown that long-term stress responses in depressed patients increase intestinal wall permeability, making it easier for aggressive bacteria or antigens to translocate to the lymphatic system or circulatory system, and then activating immune cells to trigger serum IgA and IgM production, then causing depressive episodes through systemic inflammation (Maes et al., 2008, 2012; O’Malley et al., 2010), or microbial metabolites are more easily to enter the blood circulation through the intestinal wall and pass the blood-brain barrier (BBB), directly affecting the cognitive and behavioral functions of the body (Rao et al., 2021). Notably, due to two-way communication characteristics of brain-gut axis, changes in gut microbiome composition may in turn influence depressive symptoms. Several studies have shown that the gut microbiota of people with depression differs significantly from that of people without depression in terms of diversity and abundance, with abundance being negatively correlated with the severity of depression (Jiang et al., 2015; Hu S. et al., 2019), moreover, some studies have shown that an increase in potentially harmful bacteria or a decrease in beneficial bacteria could reduce short-chain fatty acids (SCFAs) production, leading to intestinal barrier dysfunction and inflammation (Wong et al., 2006; Ramos Meyers et al., 2022). Additionally, a transplantation of patients’ gut microbiota caused mice to exhibit depression-like behavior when metabolic processes and inflammatory responses were affected by fecal microbiota transplantation (Liu et al., 2021). Hence, there may be a potential bidirectional interaction between stress and microbiome.

Antibiotics target bacteria, inhibiting their growth and proliferation, and is the most direct, widespread, and important influencing factor for changing intestinal flora composition. Antibiotics have a double-edged effect on depression, on the one hands, antibiotic treatment led to the disappearance of depression-like ethological disorders (Yu et al., 2022), and it has to be pointed out that the first drugs to be used to treat depression was Iproniazid, which was originally developed for tuberculosis (Juli and Juli, 2014; Macedo et al., 2017); on the other hands, antibiotics can damage intestinal flora’s homeostasis in the gut, resulting in depression (Hao et al., 2020). Multiple studies have indicated that antibiotic exposure increases depression risk, further, the risk of depression may increase with each additional treatment course and medication, while the declines in risk is characteristic by slow and sustained (Lurie et al., 2015; Köhler et al., 2017; Hu et al., 2022; Pouranayatihosseinabad et al., 2023).

The hypothalamic-pituitary-adrenal (HPA) axis is an important part of the neuroendocrine system. When the human body is exposed to stress, cortisol in the HPA axis is activated, which reduces inflammation and protects against extreme immune responses (Mikulska et al., 2021). However, cortisol elevation caused by chronic stress is also an important factor in the development of depression (Qin et al., 2015). Several studies have demonstrated that neuroendocrine regulation plays an important role in the pathophysiology of neuropsychiatric disorders, and there is an interaction between gut microbiota and HPA axis activity (Ge et al., 2021). Microbial communities can be changed by altering HPA axis activity [such as adrenalectomy, subcutaneous injection of adrenocorticotropic hormone (ACTH) fragments] (Amini-Khoei et al., 2019; Song et al., 2019). In addition to regulating the HPA axis dysfunction caused by stress, probiotics supplementation can also alleviate some depressive behaviors (Liang et al., 2015; Rea et al., 2016).

There are also considerable literatures suggesting a link between the vagus nerve and depression and gastrointestinal disorders (Liu et al., 2020a; Tan et al., 2022). The vagus nerve is one of the most important components of the parasympathetic system, which plays a major role in the regulation of gut-brain axis by acupuncture. Vagus nerve is a hybrid nerve with afferent and efferent fibers that senses gut microbiota metabolites and transmits information about them to the central nervous system (CNS). Additionally, activated efferent vagus nerves can also exert a systemic anti-inflammatory response by directly stimulating the HPA axis and cholinergic pathways, which alleviates damage to intestinal tight junctions and reduces intestinal permeability, thus regulating changes in microbial composition (Borovikova et al., 2000; Hu et al., 2013; Zhou et al., 2013).

## 4. Acupuncture regulates intestinal microbiota in PSD

**FIGURE 2:**
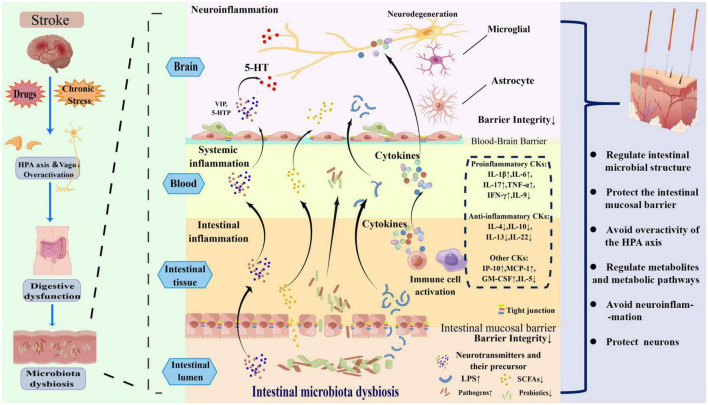
The mechanism of intestinal microbiota dysbiosis induced by post-stroke depression (PSD) and how acupuncture regulates the intestinal microbiota to treat PSD.

Once a patient has a stroke event, due to stroke, drugs, chronic stress, abnormal activation of the HPA axis and vagus nerve, a series of digestive tract symptoms will occur in the human body. These processes lead to damage to the intestinal mucosal barrier, resulting in an imbalance of intestinal microbiota due to excessive production of pro-inflammatory substances [lipopolysaccharide (LPS), proinflammatory cytokines (CKs)] and too little production of anti-inflammatory substances (SCFAs, anti-inflammatory cytokines), causing abnormal immune responses (local and systemic inflammatory responses) in the body, ultimately damaging neurons and exacerbating depression. Acupuncture can regulate the structure of intestinal microbiota, inhibit inflammatory storms and improve the symptoms of patients with PSD mainly through the following six ways in Figure 2. The original research evidence of acupuncture regulates intestinal microbiota is summarized in Table 1.

**TABLE 1:** The original researches on the regulation of intestinal microbiota by acupuncture.

No.	Acupuncture	Model	Mechanism and effect						References
			Intestinal microbial structure	Intestinal mucosal barrier	Hypothalamic-pituitary-adrenal (HPA) axis	Metabolites and metabolic pathways	Inflammatory responses	Central neurons	
1	Manual acupuncture	Female BALB/c mice [cancer-related fatigue (CRF) mice]	The abundance: Candidatus *Arthromitus*↑, *Lactobacillus*↑, Clostridia_UCG-014_unclassified↑; Escherichia–Shigella↓, Burkholderia-Caballeronia-Paraburkholderia↓, *Streptococcus*↓.	Tight junction proteins (ZO-1, Occludin, Claudin-5)↑	Restore adrenocorticotropic hormone (ACTH), corticotropin-releasing hormone (CRH) and cortisol (CORT) expression levels	Regulating the differential metabolites N-methylnicotinamide, beta-glycerophosphoric acid, geranyl acetoacetate, serotonin and phenylalanine, tyrosine and tryptophan biosynthesis, taurine and hypotaurine, and beta-alanine metabolic pathways.	In the gut and hippocampus: IL-1β↓, L-6↓, TNF-α↓.		Lv et al., 2022
2	Manual acupuncture	Male APP/PS1 mice [Alzheimer’s disease (AD)]	The abundance: *Bacteroides*↑; Firmicutes↓, Proteobacteria↓, Escherichia–Shigella↓.				In serum and brain: LPS↓, TNF-α↓, IL-1β↓.		Zhang et al., 2022d
3	Electroacupuncture	Male C57BL/6 mice [dextran sulfate sodium- (DSS-) induced colitis]	The abundance and diversity: Firmicutes↑, *Akkermansia* muciniphila↑, Bacteroidales↑, Lactobacillales↑, S24-7↑; Enterobacteriaceae↓, Proteobacteria↓, Turicibacterales↓, Erysipelotrichales↓, Turicibacteraceae↓, Clostridiaceae↓, Turicibacter↓, SMB53↓,	Tight junction proteins (Claudin-1, Occludin, ZO-1)↑		Melatonin↑, adiponectin↑, vasoactive intestinal peptide type 2 receptor (VPAC2)↑.	IFN-γ↓, TNF-α↓, IL-6↓, Th2/ILC2 related cytokines (including IL-4, IL-5, IL-9, IL-13, IL-10)↑, ILC3-derived cytokines (IL-22 and GM-CSF)↑.		Liu et al., 2020b,^2022^
4	Manual acupuncture	Male APP/PS1 mice (AD)	The abundance: *Bacteroides*↑; Proteobacteria↓, Escherichia–Shigella↓.	The damage of small intestine structures↓, tight junction proteins (Occludin and ZO-1)↑.			Lipopolysaccharide (LPS)↓, TNF-α↓.		Hao et al., 2022
5	Electroacupuncture	Hypertensive patients	Firmicutes/Bacteroidetes ratio↓. The abundance: *Blautia*↑, Escherichia–Shigella↓.						Wang J. M. et al., 2021
6	Electroacupuncture	Female Kunming mice [functional constipation (FC)]	Firmicutes/Bacteroidetes ratio↓. The relative abundance: *Roseburia*↓, *Lachnoclostridium*↓, Ruminiclostridium 9↓.						Xu et al., 2021
7	Manual acupuncture	Specific pathogen-free male Sprague–Dawley rats [chronic unpredictable mild stress (CUMS)]	*Bacteroides*/Firmicutes ratios↓. The abundance: Firmicutes↑, *Bacteroides*↓.			Dopamine (DA)↑, 5-hydroxytryptamine (5-HT)↑. Affecting the cell growth, the apoptosis pathway, the cofactor and vitamin metabolism pathway, amino acid metabolism pathway, and the carbohydrate metabolism pathway.		Enhancing brain-derived neurotrophic factor (BDNF) signaling. The mRNA and protein expression: BDNF and N-methyl-D-aspartate receptor (NMDAR)↑; Calmodulin-dependent protein kinase II-β (β-CaMKII)↓. Astrocytes↑.	Li et al., 2021
8	Electroacupuncture	Participants with knee osteoarthritis (KOA)	The abundance: *Bacteroides*↑, *Agathobacter*↑; *Streptococcus*↓.						Wang T. Q. et al., 2021
9	Manual acupuncture	Male C57BL/6 mice [Parkinson’s disease (PD)]	*Butyricimonas*↑.					Bax↓, NF-κB↓, TNF-α↓, Bcl-2↑. The activation and overexpression of microglia and astrocytes↓, Dopaminergic fibers and neurons↑.	Jang et al., 2020
10	Electroacupuncture	Male Sprague–Dawley rats [diet-induced obese knee osteoarthritis models (DIO-KOA)]	Bacteroidetes/Firmicutes ratio↑. The relative abundance of *Lactobacillus*↑. Recovery of the relative abundance of *Akkermansia*, *Clostridium*, Lactococcus, and *Butyricimonas*.			Total cholesterol↓, triglyceride (TG)↓, low-density lipoprotein (LDL)↓, high density lipoprotein (HDL)↑.	In serum: IP-10↓, IL-1α↓, MCP-1↓. In articular synovial fluid: lipopolysaccharide (LPS)↓.		Xie et al., 2020
11	Electroacupuncture	Male db/db mice (spontaneous T2DM mouse)	Probiotics: *Blautia*↑, *Lactobacillus*↑. Opportunist pathogens: *Alistipes*↓, *Helicobacter*↓, *Prevotella*↓.	Mucosal inflammation, goblet cells, and epithelial damage in the colon.		TG↓, low-density lipoprotein cholesterol (LDL-C) ↓.	SCFAs (acetic acid and butyric acid)↑, LPS↓, IL-6↓.		Zhang et al., 2021
12	Electroacupuncture	Patients with mild to moderate PD	The relative abundance: *Bacteroides*↑; *Parasutterella*↑, the genera *Dialister*↓, *Hungatella*↓, *Barnesiella*↓, *Megasphaera*↓, *Allisonella*↓, *Intestinimon*↓, *Moryella*↓.						Nazarova et al., 2022
13	Acupuncture	Rats with functional dyspepsia				Tryptophan indole metabolites			Zhang et al., 2022b
14	Electroacupuncture	Specific pathogen-free (SPF)-certified male Kunming mice [peptic ulcer disease (PUD) model]	No significant change in the alpha diversity of duodenal microbial.	The arrangement of duodenal mucosal cells was closer to normal, and the villus morphology basically returned to normal.		Trilobal factor (TFF)↑, DA↑			Li et al., 2022d
15	Electroacupuncture	Male C57BL/6J mice [high fat diet (HFD)-induced obese mice]	The abundance of jejunal and cecal microbiota was restored.			Activating Nod-like receptor signaling pathways, Defensin alpha 5 (Defα5)↑, Energy metabolism↑, Lipid metabolism↓.			Xia X. et al., 2022
16	Electroacupuncture	High fat diet (HFD)-induced obese mice	The relative abundances of gut microbiota was restored.			Regulating glycerophospholipid metabolism and primary bile acid biosynthesis.			Si et al., 2022
17	Electroacupuncture	Male Sprague–Dawley rats (HFD-induced obese rats)	Firmicutes/*Bacteroides* ratio↓. The relative abundance: *Prevotella*_9↑.			Regulating lipid metabolism and improving insulin sensitivity and glucose homeostasis.			Wang et al., 2019
18	Electroacupuncture	Ulcerative colitis (UC) mice	The diversity and abundance of gut microbiota↑.				The percentage of Treg cells in CD4+ T lymphocytes↑, the percentage of Th17in CD4+ T lymphocytes↓.		Wei et al., 2019
19	Electroacupuncture	PD mice	The abundance: Erysipelotrichaceae↓.				The mRNA levels: IL-6↓, TNF-α↓.	The loss of dopaminergic neurons↓.	Han Q. Q. et al., 2021

### 4.1. Regulation of intestinal microbial structure

Recent studies have gradually shown that acupuncture indirectly alters the microbial composition and communities in various ways, and researchers have found that post-stroke depression-like behavior is strongly associated with intestinal microbial changes after acupuncture treatment (Jiang et al., 2021).

Based on 16S rRNA sequencing, Lv et al. (2022) found that manual acupuncture treatment significantly increased the abundances of Firmicutes, Bacteroidetes, and Patescibacteria and significantly decreased the abundances of Proteobacteria in mice at the phylum level; and the abundance of Candidatus *Arthromitus*, *Lactobacillus*, Muribaculaceae_unclassified, and Clostridia_UCG-014_unclassified were significantly increased and the abundances of Escherichia–Shigella, Burkholderia-Caballeronia-Paraburkholderia, and *Streptococcus* were decreased at the genus level in response to manual acupuncture. In general, the Lv et al. (2022) study demonstrated acupuncture alleviated disease-associated gut microbiome imbalances. Furthermore, there was also a significant correlation observed between the development of depression and the content of Clostridiaceae, Candidatus *Arthromitus*, and *Lactobacillus*. Additionally, Zhang et al. (2022d) observed that manual acupuncture significantly reduced the abundance of Firmicutes, Proteobacteria and Escherichia–Shigella in Alzheimer’s disease mice, while significantly increasing the abundance of *Bacteroides*, which led to improvements in intestinal flora. Liu et al. (2020b,2022) demonstrated that electroacupuncture regulated the overall structure of the intestinal microbiota in the intestinal tract of diseased mice, making the abundance and diversity of Firmicutes is similar to what it is in the healthy mice’s intestinal tracts.

According to Hao et al. (2022), mice experiencing manual acupuncture showed a significant increase in *Bacteroides* while a decrease in Proteobacteria and Escherichia–Shigella, however, no significant improvement in intestinal microbiota diversity was found, perhaps method of calculating diversity or insufficient samples might limit the result of diversity.

Wang J. M. et al. (2021) observed how electroacupuncture affected patients’ flora structures, and observed that part of their flora structures reversed and gradually began to resemble those of healthy individuals, finding the following: at the phylum level, the relative abundance of Firmicutes and the Firmicutes/*Bacteroides* ratio decreased significantly; at the genus level, the relative abundance of *Blautia* increased while the abundance of Escherichia–Shigella decreased. And by the way, Firmicutes and *Bacteroides* are believed to be the predominant bacteria in healthy individuals’ intestinal tracts, and using the ratio between them, researchers can assess the degree of intestinal microbial health and establish a landmark parameter for determining the degree of intestinal health (Tang et al., 2019). Furthermore, *Blautia* produces a variety of SCFAs that have anti-inflammatory properties (Koh et al., 2016), and gram-negative bacteria such as Escherichia–Shigella contains LPS, which is a proinflammatory compound found in the cell wall of gram-negative bacterium (Yoo et al., 2022). Consequently, the decreased level of Escherichia–Shigella and the increased relative abundance of *Blautia* in patients treated with electroacupuncture indicate reduced host’s inflammation. Xu et al. (2021) found that electroacupuncture rebalances the structure of the intestinal microbiota in mice by reducing Firmicus/*Bacteroides* ratio and the relative abundance of *Roseburia*, *Lachnoclostridium*, and Ruminiclostridium 9 to bring them closer to healthy mice’s state. A manual acupuncture treatment was conducted on rats with depression by Li et al. (2021), and researchers found that manual acupuncture regulation could reduce *Bacteroides*/Firmicutes ratios in the intestinal tract of depressed rats and improve the biodiversity of intestinal flora. Hence, electroacupuncture and manual acupuncture are capable of reversing the proportion of gut bacteria, thus alleviating intestinal ecological disorder in patients (Liu et al., 2020c).

Wang T. Q. et al. (2021) found that electroacupuncture could reverse the increase in the abundance of *Streptococcus* in the disease state, while increasing the abundance of the *Bacteroides* and *Agathobacter* (beneficial bacteria). There was a strong correlation between fecal *Streptococcus* abundance and Hamilton depression scale (HAMD) scores (possibly related to intestinal mucosal barrier and immunity being affected by tryptophan metabolism) (Zhang et al., 2022c), Health-beneficial SCFAs can be produced by *Bacteroides* and *Agathobacter* (beneficial bacteria) in the gut that inhibit opportunistic pathogens and prevent the host from inflammation (Koh et al., 2016; Hua et al., 2020). According to Jang et al. (2020), manual acupuncture restored bacterial abundance and approximately 70% of microbiome composition in the intestinal tract of diseased mice, and increased the number of *Butyricimonas*, which has anti-inflammatory properties by increasing the production of butyrate, a SCFA (Yang et al., 2022).

Xie et al. (2020) carried out 2-week electroacupuncture intervention in mice, and found that electroacupuncture could inhibit proinflammatory shift with promoting the recovery of the relative abundance of *Akkermansia*, *Clostridium*, Lactococcus, and *Butyricimonas* in the intestinal tract, and significantly increase the relative abundance of *Lactobacillus*. A number of bacteria above are capable of inhibiting inflammation, protecting the intestinal barrier and preventing depression (Guo et al., 2022; Lai et al., 2022; Ramalho et al., 2022). All of the above bacteria have the potential to inhibit inflammation, protect the intestinal barrier, and prevent depression (Guo et al., 2022; Lai et al., 2022; Ramalho et al., 2022). It can be seen that acupuncture alleviates systemic inflammation in rats overall through the increase in beneficial microorganisms’ abundance. Zhang et al. (2021) observed that, at the phylum level, electroacupuncture could modulate the intestinal microbiota structure of T2DM mice to a level similar to that of normal control mice, and researchers found electroacupuncture could increase probiotics (*Blautia* and *Lactobacillus*) and decreased opportunist pathogens (*Alistipes*, *Helicobacter*, *Prevotella*), moreover, a significant correlation was also observed between changes in intestinal flora and changes in LDL-C. After electroacupuncture intervention for 8 weeks, Nazarova et al. (2022) observed changes in intestinal bacteria of participants, and result showed the relative abundance of *Bacteroides* and *Parasutterella* increased significantly at the genus level, whereas the abundances of the genera *Dialister*, *Hungatella*, *Barnesiella*, *Megasphaera*, *Allisonella*, *Intestinimon*, and *Moryella* were significantly lower. Thus, researchers emphasized the role of the gut-brain axis in the process of the treatment in central system diseases.

### 4.2. Regulating the intestinal mucosal barrier to prevent bacterial translocation

The composition of intestinal microbiota can be indirectly affected by acupuncture. Additionally, protecting the structure and function of the intestinal mucosal barrier system, which indirectly affects the colonization of bacteria and prevents pathogenic antigens penetrating (translocation) the physical barrier, so that human health can be maintained (Macpherson et al., 2002). As soon as the intestinal mucosal barrier is damaged, it increases permeability of the intestinal epithelium (leaky gut), which allows inflammation-related factors and other harmful substances to enter the circulatory system and initiate systemic inflammation (Wasinger et al., 2020; Dou et al., 2022). As part of the intestinal barrier, tight junctions and their proteins protect organisms from pathogens entering from the external environment, which play a significant role in maintaining intestinal barrier integrity (Lin et al., 2022). By establishing cell polarity, tight junctions determine paracellular permeability and serve as a major barrier to the paracellular pathway (Zihni et al., 2016). In intestinal epithelium, tight-junction proteins identify the permeability of paracellular ions at tight junctions, which are located mainly on the lateral sides of the junction tops of adjacent cells (Zeisel et al., 2019). As well as maintaining the integrity of the tight junctions between cells and maintaining the barrier function, it plays a role in the repair of intestinal epithelial damage (Krug and Fromm, 2020).

An experiment conducted by Lv et al. (2022) revealed that acupuncture promoted tight junction proteins (ZO-1, Occludin, Claudin-5) and improved the function of mice’s intestinal mucosal immune barriers. Additionally, this study (Lv et al., 2022) showed that intestinal tight-junction protein expression is correlated with changes in intestinal flora abundance after acupuncture intervention.

In two electroacupuncture experiments, Liu et al. (2020b,2022) found that Claudin-1, Occludin, ZO-1 properties were repaired. Thus, by improving the tight junctions of intestinal epithelial cells, it can stabilize permeability and maintain intestinal homeostasis.

In diseased animals, Hao et al. (2022) observed under an electron microscope that the damage of small intestine structures were significantly reduced after intervention with manual acupuncture. An electron-microscopical examination reveals a mild separation of the epithelium from the lamina propria, an orderly arrangement of intestinal gaps as well as narrower connection gaps. An immunofluorescence experiment reveals the fluorescence structure of tight junction proteins (Occludin and ZO-1) was restored by manual acupuncture intervention, and the fluorescence proteins showed continuity and enhanced intensity, maintaining the intestinal mucosal barrier.

### 4.3. Regulation of hypothalamic-pituitary-adrenal (HPA) axis disorders

The HPA axis disorder is closely related to the host circadian rhythm disorder and the body’s stress response. The HPA axis is regulated by the circadian rhythm cycle, and its abnormal function can trigger sleep disorders and contribute to depression development (Wirz-Justice, 2006; Kim et al., 2015). The result of a cross-sectional study examining the link between insomnia and PSD suggests that insomnia before stroke is an indicator of depression, and stroke is a risk event that can worsen depression (Zheng, 2021). In another clinical cross-sectional study, stroke survivors with poorer subjective sleep were also more likely to suffer from depression (Davis et al., 2019). Patients with PSD often suffer from sleep disorders, so the two frequently require active treatment together (Cai et al., 2021). The composition and function of the gut microbiome also exhibits circadian rhythmicity in relation to the host’s activity (Thaiss et al., 2014). This manifests itself in the fact that interference with the sleep pattern of the host can alter the expression of clock genes, ultimately altering the structure and diversity of the gut microbiome (Voigt et al., 2014; Leone et al., 2015), which in turn can drive changes in the circadian rhythm of the host (Thaiss et al., 2016). Moreover, the HPA axis, as one of the key components of stress regulation, can timely perceive pressure and quickly initiate signals in the paraventricular nucleus (PVN) of the hypothalamus, and HPA axis abnormalities may be one of the biological indicators for depression in its early stages (Spalletta et al., 2006; Du and Pang, 2015). Moreover, there is evidence that acute ischemic stroke can act as a stressor to activate the HPA axis (Wexler, 1970; Yoo et al., 2011). There are several basic studies showing that acupuncture can down-regulate the expression of CRH mRNA in hypothalamus, reduce plasma levels of ACTH and CORT (Le et al., 2016; Zheng et al., 2019), which plays an antidepressant role by inhibiting the over-excitation of HPA axis (Han X. et al., 2021). There is also a bidirectional regulatory relationship between the HPA axis and intestinal microecology. Microbiomes in the gut regulate corticosteroid production, including cortisol and glucocorticoids, in turn, the HPA axis can regulate intestinal motility and affect the living environment of intestinal microbiota, and it has been shown that overactivity of the HPA axis can increase intestinal mucosal permeability, activate intestinal immunity, and further alter the composition of microbiome in the intestines, disrupting the gut-microbiome balance (Li et al., 2018; Wu et al., 2018; Młynarska et al., 2022).

According to Lv et al. (2022), manual acupuncture could restore ACTH, CRH and cortisol CORT expression levels, as well as improve dysfunction of the HPA axis. Also, this study found that changes in intestinal flora abundance and hormone expression were correlated after manual acupuncture intervention, suggesting that the regulation of the HPA axis by acupuncture is related to acupuncture’s influence on the intestinal flora composition.

### 4.4. Effect on metabolites and metabolic pathways

There are 100 times more genes in the gut microbiome than in the human genome, and those genes can encode at least 10 times as many unique genes as the host’s genes (Ley et al., 2006). It is likely that the products of these genes play an important role in the pathogenesis of depression after entering the circulation and integrating into the host metabolic pathway (Li et al., 2023). Moreover, as a consequence of stroke, the structural integrity of the BBB is affected and under inflammatory conditions, matrix metalloproteinases (MMPs) can degrade basal layer proteins, increasing the BBB’s permeability (Zlokovic, 2006; Lakhan et al., 2013). LPS, SCFAs, adiponectin, vasoactive intestinal peptide (VIP), and some neurotransmitter precursors (e.g., 5-HTP) were more readily transported across the BBB to the brain due to increased BBB permeability (Birdsall, 1998; Dogrukol-Ak et al., 2003; Nedorubov et al., 2019; Megur et al., 2020; Formolo et al., 2022; Zhao et al., 2022).

Multiple metabolism pathways and metabolites were altered by manual acupuncture in subjects according to Lv et al. (2022). A serum metabolomics study conducted by Lv et al. (2022) has revealed the following: acupuncture can regulate the differential metabolites, including biosynthesis of N-methylnicotinamide, beta-glycerophosphoric acid, geranyl acetoacetate, serotonin and phenylalanine, tyrosine and tryptophan, as well as metabolic pathways of hypotaurine and beta-alanine taurine and hypotaurine, and beta-alanine. And it should be noted that the metabolic pathways and metabolites described above are closely associated with multiple neurotransmitter precursors of depression-related (Parker and Brotchie, 2011; Strasser et al., 2016; Hüfner et al., 2019). As a result of Lv et al. (2022) correlation analysis of differential microflora and differential metabolites, the authors speculated that the changes of microflora caused by manual acupuncture will affect the changes in serum metabolites, and integrating acupuncture into the process of regulating depression.

It has been reported that the intestinal microbiota affects neurotransmitter production and tryptophan metabolism (O’Mahony et al., 2015), and that tryptophan can produce a variety of indole metabolites under the influence of the microbiome. In the intestinal environment, tryptophan and its indole metabolites are precursors or signaling molecules of many bioactive substances (such as 5-HT, Aryl -Hydrocarbon, Oxindole and Isatin), which have an important role to play in the “gut-brain axis” (Hubbard et al., 2015; De Vadder et al., 2018; Jaglin et al., 2018; Roager and Licht, 2018; Qu et al., 2019; Li et al., 2020; Zhang et al., 2022c). By chemical labeling assisted liquid chromatography-tandem mass spectrometry, Zhang et al. (2022b) successfully determined 15 tryptophan indole metabolites in feces of rats with functional dyspepsia after acupuncture intervention.

Manual acupuncture treatment was administered to depressed rats by Li et al. (2021), and his results showed that the levels of DA and 5-HT in serum and hippocampus increased after treatment. After Kyoto Encyclopedia of Genes and Genomes (KEGG) analysis, it was found that manual acupuncture affected the cell growth, the apoptosis pathway, the cofactor and vitamin metabolism pathway, amino acid metabolism pathway, and the carbohydrate metabolism pathway in rats, as well as improving their depression-like behavior through the brain-gut axis.

Li et al. (2022d) detected the diversity and richness of microflora in the stomach and duodenum after electroacupuncture, as well as the content changes of VIP, DA, and trilobal factor (TFF) in serum. Researchers found that electroacupuncture increased TFF and DA levels in the serum, as well as the diversity and richness of stomach microbiota. In addition to being protective to gastrointestinal mucosa (Mashimo et al., 1996; Gyires, 2004; Huang and Wu, 2021), trefoil factor can also reverse depression-like behavior in the same way as dopamine (Shi et al., 2012; Li et al., 2015). Accordingly, electroacupuncture’s effectiveness may be related to levels of dopamine and trefoil factor and microbiome structural changes.

Adiponectin, as a adipocyte derived protein, can inhibit the infiltration of macrophages and the increase of pro-inflammatory cytokines, maintain intestinal homeostasis and improve intestinal barrier integrity (Obeid et al., 2017), and is significantly associated with Firmicutes differential OTUs, which may be a key node between intestinal microbiota and depression (Bai et al., 2022). Liu et al. (2020b,2022) found electroacupuncture restored melatonin and adiponectin levels in plasma to near-normal levels in diseased rats as a result of electroacupuncture, while it could also restore the expression of VIP and its VIP type 1 receptor (VPAC1) and its VIP type 2 receptor (VPAC2). In addition, there is evidence that VIP can improve the immunity of intestinal mucosa (Seillet et al., 2020), and it is also associated with biological depression (Yu et al., 2021; Shukla et al., 2022), thus, acupuncture may be a mediator of gut-brain communication.

According to studies of Xia X. et al. (2022), electroacupuncture activated Nod-like receptor signaling pathways and promoted intestinal defensin production in order to protect the host from intestinal pathogens, thus maintaining intestinal homeostasis. Based on Spearman correlation coefficient analysis, Xia X. et al. (2022) suggested that electroacupuncture of intestinal defensins appeared to be a key mechanism for restoring intestinal microflora homeostasis. Furthermore, electroacupuncture can also upregulated energy metabolism and down-regulate lipid metabolism.

Si et al. (2022) found that electroacupuncture intervention restored 10 significantly altered bacterial genera and 11 metabolites in obese mice to normal levels, as well as that intestinal flora and metabolic levels were strongly correlated. According to researchers’ speculation, acupuncture restored gut flora balance primarily by regulating glycerophospholipid metabolism and primary bile acid biosynthesis. Several studies have shown that intestinal microflora is involved in the pathogenesis of depression through glycerophospholipid metabolism and primary bile acid biosynthesis (Gong et al., 2021; MahmoudianDehkordi et al., 2022). Accordingly, it can be speculated that electroacupuncture might be useful in treating depression by regulating intestinal flora’s production of glycerophospholipids and bile acids.

The study of Xie et al. (2020) found that electroacupuncture could reduce the level of total cholesterol, TG and LDL in serum, while improving the level of HDL. This result may be related to the increase of the relative abundance of *Lactobacillus*, thus affecting lipid metabolism. And according to relevant study, the aberrant lipid metabolism is one of the predictive biological indicators of PSD (Zhong et al., 2022).

Wang et al. (2019) showed that electroacupuncture could regulate lipid metabolism and improve insulin sensitivity and glucose homeostasis by regulating intestinal flora composition (mainly by reducing Firmicutes/*Bacteroides* ratio and increasing *Prevotella*_9 abundance).

### 4.5. Effect on inflammatory responses

During times of inflammation or homeostasis disorders, the microbiota can act as a protective force for the body by affecting the immune system’s function. Basically, the gut microbiota protects host by controlling the function and number of inflammatory cells, either directly or indirectly, in response to systemic or local infection challenges (Gaboriau-Routhiau et al., 2009; Ivanov et al., 2009; Miller et al., 2009). Certainly, there may also be the overabundance of bacteria in the intestinal tract which have potential to magnify inflammation, leading to local and systemic pathological consequences effects (Belkaid and Hand, 2014). According to the regulation effect of acupuncture, on the one hand, acupuncture can restore the balance of intestinal flora structure, adjust the proportion, abundance and number of pathogenic bacteria and beneficial microorganisms, thus affecting the activation or inhibition of pro-inflammatory and anti-inflammatory cells. On the other hand, acupuncture can protect the structure and function of intestinal mucosal barrier system, prevent the translocation of pathogenic bacteria and inflammation-causing substances, thereby avoiding the occurrence of inflammatory storms in the body.

Basic studies have shown that increased production of proinflammatory cytokines after cerebral ischemia can activate indoleamine 2,3-dioxygenase (IDO) in glial cells and reduce the bioavailability of tryptophan (tryptophan is metabolized mainly *via* two main pathways, the serotonin and kynurenine pathways), as a result, 5-HT is depleted, serotonergic transmission is blocked, as well as neuroactive tryptophan metabolites (such as kynurenine) are produced (O’Connor et al., 2009; Souza et al., 2017; Körtési et al., 2022), which eventually leading to PSD (Spalletta et al., 2006). Despite the lack of a complete understanding of the pathophysiology of depression, inflammation is a key driver of its development, and inflammatory factors is important biological factors that increase the risk of depressive episodes. Several cohort studies have found that the increase of serum levels of proinflammatory factors (such as IL-6, IL-17, TNF-α, and IL-1β) in the acute phase after stroke is independent predictors of depression when using logistics regression analysis (Kim et al., 2017; Hu J. et al., 2019), and reducing the expression of IL-6, TNF-α, and IL-1β in the cortex and hippocampus alleviated depression-like behavior in rats with PSD (Yan et al., 2019).

Post-stroke depression is a brain disease. In addition to stroke itself, which activates glial cell activation and causes CNS inflammation (Lubart et al., 2021; Rayasam et al., 2022; Tariq et al., 2022), peripheral inflammatory factors may establish relationship with the CNS after crossing the BBB as well (Beurel et al., 2020). Furthermore, a significant correlation between CNS inflammation and peripheral inflammation is also supported (Leng et al., 2018; Richards et al., 2018). It is worth noting that inflammation of the CNS is closely associated with microglia and astrocytes, and there has been a lot of evidence that inflammatory microglia and astrocytes play an important role in the development of depression (Rajkowska and Stockmeier, 2013; Peng et al., 2015; Leng et al., 2018; Xia W. et al., 2022; Xie et al., 2022).

As soon as intestinal barrier function is compromised, the bacterial translocation becomes easy, the immune system becomes activated and inflammatory factors increase, resulting in almost all of the changes associated with depression occurring in neural activity (such as neuroendocrine function, neuroplasticity, neurotransmitter signaling, cerebrovascular endothelial cell signaling, circumventricular organ signaling, and peripheral immune cell-to-brain signaling and so on), eventually, neuroinflammation causes behavioral changes and depression (Smith, 1991; Miller et al., 2009; Miller and Raison, 2016; Kronsten et al., 2022).

Lv et al. (2022) found that manual acupuncture inhibited the levels of pro-inflammatory cytokines (IL-1β, IL-6, and TNF-α) in the gut and hippocampus, and there was a correlation analysis that suggested that acupuncture promoted intestinal microbiota regulation, improved intestinal barrier function, reduced intestinal inflammation, and decreased central inflammation.

Pro-inflammatory cytokines (TNF-α and IL-1β) and LPS may induce depressive symptoms and are the most reliable biomarkers for the presence of inflammation in depressed patients (Spalletta et al., 2006; Maes et al., 2008; Miller et al., 2009). Researchers have proven that intestinal barrier destruction in depression is related to an increase in proinflammatory factors like LPS and TNF-α and IL-1β (Guo et al., 2022). As a consequence of acupuncture, significant reductions in LPS, TNF-α, and IL-1β in serum and brain were observed, and Zhang et al. (2022d) hypothesized that this was the result of manual acupuncture restoring the intestinal barrier and reducing inflammation by regulating the intestinal flora.

Xie et al. (2020) examined the inflammatory cytokines and inflammatory mediators in serum and articular synovial fluid of rats, and the results showed that electroacupuncture could reduce the levels of IP-10, IL-1α, and MCP-1 in serum and LPS in articular synovial fluid, and play an anti-inflammatory role.

Hao et al. (2022) observed that after manual acupuncture intervention, the fluorescence intensity of LPS that could be stained by immunofluorescence decreased, as well as the number of cells that could express glial fibrillary acidic protein (GFAP) in the lamina propria of the intestine and the contents of LPS and TNF-α in serum and intestinal tract. Researchers suggested by reducing the toxic effect of TNF-α on intestinal mucosa and the inflammatory effect of LPS, the structure of tight junction proteins was protected, and inflammatory mediators were reduced into the circulation, thus protecting the CNS.

It has been found that SCFAs, which are metabolized by the gut microbiome, are associated with changes in the gut microbiome in depressed mice (Hao et al., 2022). Electroacupuncture was studied to determine its effect on serum SCFA content, and Ke et al. (2022) found a strong correlation between prognosis of apoplexy rats and intestinal microbiota production of SCFAs (especially acetic acid and propionic acid). Electroacupuncture may improve stroke outcomes by increasing acetic acid and propionic acid levels to restore energy supply to the intestinal epithelium, reduce intestinal inflammation, and stabilize intestinal microbiota. The study of Zhang et al. (2021) showed that the concentration of SCFAs (acetic acid and butyric acid) could be increased in feces as a result of electroacupuncture, which may be related to an increase in *Lactobacillus* and *Blautia*. The study of Zhang et al. (2021) also showed that significantly reduced serum levels of inflammation markers such as LPS and IL-6, and positively correlated with changes in population of *Alistipes*, *Helicobacter* and *Prevotella*, and histopathological analysis revealed that there was significantly less mucosal inflammation, goblet cells, and epithelial damage in the colon.

It is believed that intestinal epithelial cells contain a variety of pattern recognition receptors, including toll-like receptors (TLRS), that are important for the regulation of inflammatory responses by invading pathogens and pathogen-produced toxins (Chassin et al., 2010; Belkaid and Hand, 2014). In their previous experiment, Liu et al. (2020b) discovered that electroacupuncture could inhibit the proinflammatory factors IFN-γ, TNF-α, and IL-6 through TLR4 signaling *via* MyD88-dependent pathway to prevent excessive immune response in the whole body. According to subsequent related experiments, Liu et al. (2022) also found that electroacupuncture could reduce the level of proinflammatory factor IL-6 in plasma, and significantly increase Th2/ILC2 related cytokines (including IL-4, IL-5, IL-9, IL-13, IL-10), as well as increase ILC3-derived cytokines IL-22 and GM-CSF, among which IL-10 is a potent anti-inflammatory cytokine for ILC2s to exert their functions.

Regulatory T cells (Treg) and pro-inflammatory T helper T cell 17 (Th17) cells are a pair of CD4+ T lymphocyte subsets that are functionally opposite, with Th17 promoting tissue inflammation while Treg exhibiting anti-inflammatory properties, and PSD is driven in part by an imbalance between the two cell subsets forming the immune axis (Ju and Wang, 2019; Cui et al., 2021; Westfall et al., 2021). Depressive symptoms can be improved by regulating the gut microbiome’s role in regulating the Treg/Th17 immune axis (Westfall et al., 2021). In their study, Wei et al. (2019) found that electroacupuncture regulated the increase in the diversity and abundance of gut microbiota, positively correlated with the improvement in the percentage of Treg cells in CD4+ T lymphocytes, and negatively correlated with the percentage of Th17, indicating that a possible mechanism by which electroacupuncture may regulate gut microbiota structure is through its effects on the internal immune environment.

### 4.6. Regulation of central neurons

In stroke survivors, depression is associated with survival status of neurons (Yang et al., 2018; Zavvari and Nahavandi, 2020). The findings of Han Q. Q. et al. (2021) suggested that electroacupuncture can reduce the abundance of Erysipelotrichaceae that have pro-inflammatory properties, decrease the mRNA levels of proinflammatory cytokines IL-6, TNF-α and reduce the loss of dopaminergic neurons in the substantia nigra (SN). Researchers speculated that electroacupuncture acted as a neuroprotective role on dopaminergic neurons by inhibiting inflammation in the SN to alleviate behavioral defects in mice, and this effect may be related to the regulation of intestinal microbes. Jang et al. (2020) suggested that the immunomodulatory function of the gut microbiome plays a key role in the process of neuroprotection and anti-inflammation. Manual acupuncture can inhibit the expression of Bax, NF-κB and TNF-α and restore the expression of Bcl-2, and reduce the activation and overexpression of microglia and astrocytes. Neuroprotection occurs through manual acupuncture by blocking neuroinflammation responses and apoptosis, and increasing the level of dopaminergic fibers and neurons in the striatum and SN. Li et al. (2021) conducted manual acupuncture treatment on depressed rats and they found that acupuncture regulates gut microbes and neurotransmitters to alleviate depression-like manifestations in rats. Brain-derived neurotrophic factor (BDNF) signaling was enhanced by manual acupuncture intervention, increasing the mRNA and protein expression of BDNF and N-methyl-D-aspartate receptor (NMDAR), and increasing the number of astrocytes in the hippocampus as a result, at the same time, the mRNA and protein expression of β-CaMKII, which can block BDNF receptor, was decreased in the hippocampus.

## 5. Conclusion and prospects

Acupuncture can positively promote the prognosis of patients with PSD by maintaining the dynamic balance of intestinal flora structure, which proves that acupuncture is a promising non-drug treatment for reducing depressive symptoms. This paper examines the relationship between intestinal flora and PSD and the role of acupuncture in this relationship to summarize acupuncture will be able to treat PSD through multiple targets (Protect the intestinal mucosal barrier system, avoid overactivity of the HPA axis to activate intestinal immunity, regulate metabolites and metabolic pathways to maintain intestinal homeostasis, control the balance of inflammatory cells and inflammatory factors, avoid neuroinflammation and protect central neurons), and proposes that the common and core link of these mechanism is that intestinal microbiota regulates the local and systemic immune system.

However, the above studies involving acupuncture and intestinal flora structure adjustments are only capable of proving correlation rather than causation. From the current development perspective of acupuncture in the treatment of PSD, the mechanism of acupuncture to maintain the dynamic balance between the type and number of intestinal flora to treat and prevent depression has not been thoroughly studied. And it remains a major challenge to understand the dynamics of microbial ecological adjustment *in vivo*.

The future should involve more researches to explore whether acupuncture can restore ecological balance of intestinal microbes in the immune deficiency model of depression to improve depression, and determine the causal relationship among the three to fill the gaps in current knowledge. It is believed that with more studies, the pathogenesis of depression will be further clarified in the future.

## Author contributions

HJ: conceptualization and writing—original draft. SD and JZ: writing—review and editing. BL, WZ, JC, and MZ: investigation. ZM: supervision. CZ: project administration. All authors read and agreed to the published version of the manuscript.
